# Gestational diabetes and the human salivary microbiota: a longitudinal study during pregnancy and postpartum

**DOI:** 10.1186/s12884-020-2764-y

**Published:** 2020-01-31

**Authors:** Mie K. W. Crusell, Lærke R. Brink, Trine Nielsen, Kristine H. Allin, Torben Hansen, Peter Damm, Jeannet Lauenborg, Tue H. Hansen, Oluf Pedersen

**Affiliations:** 1 Novo Nordisk Foundation Center for Basic Metabolic Research, Section of Human Genomics and Metagenomics in Metabolism, University of Copenhagen, Blegdamsvej 3B 8th floor, 2200 Copenhagen N, Denmark; 2 Department of Gynecology and Obstetrics, Hvidovre University Hospital, Kettegaardsvej 30, 2650 Hvidovre, Denmark; 3 Center for Clinical Research and Prevention, Bispebjerg and Frederiksberg Hospital, the Capital Region, Nordre Fasanvej 57, 2000 Frederiksberg, Denmark; 4 Department of Obstetrics, Center for Pregnant Women with Diabetes, Rigshospitalet University Hospital, Blegdamsvej 9, 2100 Copenhagen Ø, Denmark; 5 Institute of Clinical Medicine, Faculty of Health and Medical Sciences, University of Copenhagen, Blegdamdsvej 3B, 2200 Copenhagen N, Denmark; 6 Department for Gynecology and Obstetrics, Herlev University Hospital, Herlev Ringvej 75, 2730 Herlev, Denmark; 7 Department of Cardiology and Endocrinology, Slagelse Hospital, Ingemannsvej 30, 4200 Slagelse, Denmark

**Keywords:** Salivary microbiota, Gestational diabetes mellitus, Gestational hyperglycaemia, Pregnancy, Glycaemic traits, Bacterial species

## Abstract

**Background:**

An aberrant composition of the salivary microbiota has been found in individuals with type 2 diabetes, and in pregnant women salivary microbiota composition has been associated with preeclampsia and pre-term birth. Pregnant women, who develop gestational diabetes (GDM), have a high risk of developing type 2 diabetes after pregnancy. In the present study we assessed whether GDM is linked to variation in the oral microbial community by examining the diversity and composition of the salivary microbiota.

**Method:**

In this observational study the salivary microbiota of pregnant women with GDM (*n* = 50) and normal glucose regulation (*n* = 160) in third trimester and 9 months postpartum was assessed by 16S rRNA gene amplicon sequencing of the V1-V3 region. GDM was diagnosed in accordance with the International Association of the Diabetes and Pregnancy Study Groups (IADPSG) criteria. Cross-sectional difference in alpha diversity was assessed using Student’s t-test and longitudinal changes were assessed by mixed linear regression. Cross-sectional and longitudinal difference in beta diversity was assessed by permutational multivariate analyses of variance. Differentially abundant genera and OTUs were identified by negative binomial regression.

**Results:**

In the third trimester, two species-level operational taxonomic units (OTUs), while eight OTUs postpartum were differentially abundant in women with GDM compared with normoglycaemic women. OTU richness, Shannon diversity and Pielou evenness decreased from late pregnancy to 9 months after delivery regardless of glycaemic status.

**Conclusion:**

GDM is associated with a minor aberration of the salivary microbiota during late pregnancy and postpartum. For unknown reasons richness of the salivary microbiota decreased from late pregnancy to postpartum, which might be explained by the physiological changes of the immune system during human pregnancy.

## Background

The human oral cavity harbors a complex ecosystem of hundreds of different microbes primarily as biofilms on the surfaces of the teeth [1]. The healthy oral microbiota is in a symbiotic state with its host, but dysbiosis of the oral microbial community often results in development of dental disease [2, 3]. Increasing evidence suggests that the oral microbiota is able to migrate to extra-oral habitats, causing inflammation [4]. Epidemiological studies have linked periodontal disease to obesity [5], insulin resistance [6] and type 2 diabetes [7]. In a rodent study, oral administration of *Porphyromonas gingivalis*, a periodontal pathogen, induced systemic inflammation and insulin resistance [8]. A meta-analysis found that periodontal treatment in type 2 diabetes patients improved the glycaemic regulation in a 3 months follow-up period [9]. All together, the available literature provides suggestive evidence of a role of oral microbiota in the pathophysiology of metabolic disorders.

Gestational diabetes mellitus (GDM) is one of the most common obstetric complications, characterized by hyperglycaemia during pregnancy [10]. Gestational diabetes is associated with adverse pregnancy outcomes [11, 12] and has long-term consequences for both the women and their offspring [11–15]. Like GDM, periodontal disease has been linked to adverse pregnancy outcomes such as preterm birth [16], low birth weight [16, 17] and pre-eclampsia [18–20], suggesting a possible involvement of the oral microbiota in GDM development.

Interestingly, evidence suggest similarities between the placental and oral microbiota [21] and recent reports indicate that women with GDM have an altered placental microbiota compared with normoglycaemic women [22, 23]. In a murine model of translocation of oral bacterial species, human salivary microbiota injected into mice was identified in the placenta, with higher abundance compared with the original abundance in the oral cavity [24]. If the salivary microbiota is influenced by GDM it might contribute to the future increased risk of disorders in the offspring by affecting the placental microbiota and may likewise be a potential causal factor for the increased risk of later metabolic disorders in women who have suffered from GDM.

In the present study we assessed whether GDM is linked to variation in the oral microbial community by examining the diversity and composition of the salivary microbiota in third trimester of pregnancy and about 9 months postpartum.

## Methods

### Study population and design

The study design, clinical examination and characteristics of the participants have been described in details previously [25]. In brief, a total of 213 pregnant women in third trimester (27–33 gestational weeks) were included in the study. They women were referred to a 2-h 75 g oral glucose tolerance test (OGTT) based on the presence of one or more risk factors for GDM (a family history of type 2 diabetes mellitus; a previous delivery of a child weighing ≥4500 g at birth; glucosuria; a pre-pregnancy BMI ≥27 kg/m^2^; a known polycystic ovarian syndrome). They all volunteered and fulfilled the inclusion criteria of being singleton pregnant (verified by an ultrasound scan), of Danish ethnicity and without preeclampsia at time of inclusion. Exclusion criteria were GDM in a previous pregnancy and use of antibiotics within the last 2 months. All included participants were re-invited for a follow-up visit on an average of 9 months after delivery.

Anthropometric measurements were recorded at both time points and participants completed a questionnaire on health and lifestyle, physical activity and a validated food frequency questionnaire (FFQ) [26]. Information about gestational age at previous birth, mode of delivery and antepartum, intrapartum or postpartum antibiotics treatment was obtained from hospital birth records.

### Biochemistry and derived traits

Venous blood for measurement of hormonal and metabolic biomarkers was collected after a 10-h overnight fast. A 75 g 2-h OGTT with 75 g of glucose dissolved in 200 ml of water and sampling of venous blood at 0, 30, 60 and 120 min was conducted.

Homeostasis model assessment of insulin resistance (HOMA-IR) was calculated as (Glucose _0 min_ × Insulin _0 min_)/22.5., and whole-body insulin sensitivity (ISI) was calculated according to Matsuda et al. [27]. Insulinogenic index (IGI) and disposition index (DI) were calculated as ΔInsulin _0–30 min_ / ΔGlucose _0–30 min_ and IGI/ISI, respectively.

GDM was diagnosed according to the International Association of the Diabetes and Pregnancy Study Groups (IADPSG) criteria: fasting plasma glucose ≥5.1 mmol/L and/or 1-h plasma glucose ≥10.0 mmol/L and/or 2-h plasma glucose ≥8.5 mmol/L. [28]

### Microbiome analyses

#### DNA extraction, library preparation, sequencing and initial preparation of data

Saliva samples were collected unstimulated in a sterile tube after an overnight fast without tooth brushing at the day of the OGTT. After collection, the salivary samples were immediately frozen on dry ice and stored at − 80 °C until DNA extraction. Two-hundred-twelve women provided saliva samples in the third trimester, and 125 also provided a saliva sample 9 month after delivery.

The NucleoSpin Soil kit (Macherey-Nagel) was used to extract total genomic DNA from 2 ml of saliva. The saliva was suspended in SL2 buffer containing SX enhancer and cell disruption was performed by bead beating for 3 min at 30 Hz using a TissueLyser (Qiagen).

Variable regions V1-V3 of the 16S rRNA gene were amplified using 8F/518R fusion primers, followed by pooling and paired-end sequencing on an Illumina MiSeq platform (PE300), producing a total of 22,073,412 (median 66,154; range 19,084 – 166,564) reads. Processing of raw sequence data included truncation of sequence reads not having an average quality of 20 over a 30 bp sliding window based on the phred algorithm; removal of trimmed reads having less than 75% of their original length; removal of reads with adapter contamination (15 bases overlapped by reads and adapter with maximal 3 base mismatch allowed); removal of reads with ambiguous nucleotides; removal of reads with low complexity (reads with 10 consecutive identical nucleotides). Due to low quality of the reverse sequences, the majority of paired reads failed to overlap; hence, only forward reads were carried forward for analysis. Reads were clustered to Operational Taxonomic Units (OTUs) by 97% similarity using USEARCH (v7.0.1090) [29] and chimeras were removed using UCHIME (v4.2.40). OTU representative sequences were taxonomically classified using the Ribosomal Database Project (RDP) Classifier v.2.2 with the RDP database (Release 9) as reference. Unassigned or non-bacterial OTUs were removed leaving a total of 8,470,450 (median 25,238; range 17,641 – 27,395) sequences available for analysis. A phylogenetic tree of OTU representative sequences was constructed using FastTree (v1.0).

The final dataset of third trimester pregnant women consisted of 212 samples; 50 from women with GDM, 160 from normoglycaemic women, and two from women with uncertain diabetes status due to missing information about fasting plasma glucose) and 125 postpartum samples (43 and 81 from previous GDM and normoglycaemic women, respectively).

### Statistical analyses

R version 3.4.2 (www.r-project.ort) was used for statistical analyses. Student’s t-test and *χ*^*2*^-test were used to test for differences in clinical characteristics for continuous and categorical variables respectively. To improve normality and homoscedasticity continuous variables were log transformed, if required. Data that were non-normally distributed were tested using a Wilcoxon signed-rank test. Dietary intake during pregnancy and postpartum was compared by mixed linear regression with a random effect of individual*.*

Analyses of microbiota features were performed as previously described [25]. In brief OTU richness and Shannon’s diversity index were calculated based on rarefied OTU counts using the *phyloseq* R package. Pielou’s evenness index was calculated as Shannon’s index/log_e_(richness). Student’s t-test was used to analyse cross-sectional difference in alpha diversity between groups during pregnancy and postpartum. Change in OTU richness, Shannon’s diversity and Pielou’s evenness index from pregnancy to postpartum was assessed using mixed linear models with a random effect of subject. A model with a two-way interaction between GDM status and time (factor with four levels) as the independent variable was fitted to asses differential change in alpha diversity metrics from pregnancy to postpartum in women with and without GDM, using a post hoc t-test to contrast the difference between groups postpartum minus the difference during pregnancy. Restricted maximum likelihood was used for model fitting. Residual plots and normal probability plots were inspected to check model assumptions.

The *vegan* R packages were used for analyses of community structure*.* Permutational analysis of variance (PERMANOVA) of weighted UniFrac distances was used to assess cross-sectional difference in community structure between women with and without GDM and difference in community structure between women with GDM diagnosed by either fasting hyperglycaemia or glucose stimulated hyperglycaemia or a combination of the two. PERMANOVA of weighted UniFrac distances was also used to assess relationship between glycaemic traits and community structure. The *capscale* function of the vegan package was used to perform principal coordinate ordination. The *envfit* function was used to fit vectors of glycaemic traits onto the ordination. PERMANOVA models were fitted with permutations constrained within each individual for longitudinal analyses of community structure. By contrasting the levels of the interaction between GDM status and time, as described above, differential change in community structure from pregnancy to 9 months after delivery in women with and without GDM was assessed.

Cross-sectional differences at OTU and genus level, were assessed by negative binomial regression of unrarefied, untransformed OTU counts as implemented in the *DESeq2* R package [30]. Only OTUs with more than 2 reads in at least 10% of samples (333 of 613 OTUs) were considered.

To test the association of individual genera and OTUs with glycaemic traits discretized by a tertile split, a negative binomial Wald test as implemented in the *DESeq2* package was applied, contrasting the upper and lower tertiles of each trait, with and without adjustment for BMI.

Only results significant at a 10% false discovery rate according to the Benjamini-Hochberg method are reported.

## Results

### Clinical characteristics of the study group

Clinical characteristics of the participating women have been reported previously [25]. In brief, women with GDM were insulin resistant and showed other signs of dysmetabolism, as expected (Table 1). The two groups were comparable in age, height and duration of pregnancy, as well as in overall dietary intake based on distribution of macronutrients and intake of multivitamin supplements (Table 1, Additional file 1: Table S1, Table S2). No women had artificial tooth implants, complains of mouth dryness or periodontal complains. The periodontal status was not assessed further. A total of 125 women were re-examined at an average of 9 months after delivery, where plasma glucose and insulin concentrations were significantly higher during OGTT and Matsuda index of insulin sensitivity was decreased in women with preceding GDM (*n* = 43) compared with women with a preceding normoglycaemic pregnancy (*n* = 81) (Table 2, Additional file 1: Table S3). Markers of lipid metabolism, inflammation and breastfeeding were comparable between the two groups of women (Table 2, Additional file 1: Table S3). The women that participated in the follow-up examinations were comparable in terms of age, BMI and parity with the women that were lost to follow-up.

**Table 1:** Clinical variables of women with GDM and normal glucose regulation in third trimester pregnancy

	GDM (*n* = 50)	Normoglycaemic (*n* = 161)	*P*
Descriptive measurements			
Age (years)	34.4 (4.4)	33.3 (4.6)	0.1
Gestational age at examination (weeks)	28.7 (1.4)	28.4 (1.1)	0.1
Systolic BP (mmHg)	118 (11.2)	111 (10.6)	0.001
Diastolic BP (mmHg)	71 (8.1)	67 (8.5)	0.001
Height (cm)	168.6 (6.6)	169.7 (5.6)	0.2
Weight (kg)	91.8 (14.1)	87.3 (14.6)	0.06
Pre-pregnancy weight (kg) (*n* = 190)	83.5 (16.0) (*n* = 43)	78.5 (15.2) (*n* = 145)	0.06
Pre-pregnancy BMI kg/m^2^(*n* = 189)	29.3 (5.6) (*n* = 43)	27.1 (4.8) (*n* = 144)	0.02
Weight gain (kg) (*n* = 188)	7.4 (4.7) (*n* = 43)	8.8 (7.5) (*n* = 145)	0.3
Biochemistry at 75 g OGTT			
Plasma glucose at time 0 min (mmol/L)	5.2 (0.4)	4.6 (0.2)	5.8 × 10^−31^
Plasma gucose at time 30 min (mmol/L)	8.1 (1.0)	6.9 (0.9)	2.7 × 10^−12^
Plasma glucose at time 60 min (mmol/L)	8.9 (1.3)	7.1 (1.2)	1.9 × 10^− 15^
Plasma glucose at time 120 min (mmol/L)	7.9 (1.4)	6.2 (1.0)	5.0 × 10^−17^
Plasma insulin at time 0 min (pmol/L)	112.1 (41)	82.7 (40)	3.3 × 10^−5^
Plasma insulin at time 30 min (pmol/L)^a^	509 (228)	513 (349)	0.7
Plasma insulin at time 60 min (pmol/L)^a^	743 (476)	569 (448)	0.058
Plasma insulin at time 120 min (pmol/L)^a^	769 (640)	479 (320)	6.3 × 10^− 6^
HbA1c (mmol/mol)	34.0 (3.1)	33.0 (2.8)	0.02
HOMA-IR	2.1 (0.8)	1.5 (0.7)	1.3 × 10^− 6^
Matsuda index	3.5 (1.5)	5.3 (1.6)	3.0 × 10^− 8^
Disposition index	4.1 (1.6)	7.8 (1.6)	9.2 × 10^− 12^
Insulinogenic Index	1.2 (1.6)	1.5 (1.6)	0.004
Plasma hsCRP (mg/L)	4.4 (1.9)	3.8 (2.2)	0.2

Data presented as mean (standard deviation) or as ^a^median (interquartile range). For continuous variables P was calculated by two-tailed t-test and for categorical variables P was calculated by chi square test or Fisher’s exact test. *BMI* Body Mass Index, *BP* Blood pressure, *GA* Gestational age, *HbA1c* Glycated haemoglobin, *HOMA-IR* Homeostatic model of insulin resistance, *hsCRP* High sensitive C-reactive protein

**Table 2:** Clinical variables of GDM and normoglycaemic women 9 months after delivery

	Previous GDM (*n* = 43)	Previous normoglycaemic (*n* = 82)	*P*
Descriptive measurements			
Systolic BP (mmHg)	120 (11.2)	116 (8.7)	0.04
Diastolic BP (mmHg)	79 (8.2)	75 (8.0)	0.03
BMI (kg/m^2^)	30.0 (5.9)	29.2 (5.0)	0.4
Weight (kg)	85.3 (16.9)	84.6 (15.4)	0.8
Fat (%)	41.3 (6.6)	40.2 (6.4)	0.3
Biochemistry			
Plasma glucose at time 0 min (mmol/L) (*n* = 114)	5.4 (0.4) (*n* = 41)	5.1 (0.3) (*n* = 73)	3.7 × 10^−5^
Plasma glucose at time 30 min (mmol/L)	7.7 (1.1)	7.0 (1.0)	0.001
Plasma glucose at time 60 min (mmol/L)	7.7 (1.9)	6.6 (1.4)	0.004
Plasma glucose at time 120 min (mmol/L)	6.5 (1.2)	6.0 (1.0)	0.025
Plasma insulin at time 0 min (pmol/L)^a^	62 (56)	60 (41)	0.1
Plasma insulin at time 30 min (pmol/L)^a^	384 (180)	316 (227)	0.05
Plasma insulin at time 60 min (pmol/L)^a^	463 (373)	290 (269)	0.004
Plasma insulin at time 120 min (pmol/L)^a^	288 (204)	246 (170)	0.05
HOMA2-IR	1.4 (0.7)	1.3 (0.9)	0.4
Matsuda Index	5.8 (1.6)	7.6 (1.8)	0.01
Disposition Index	6.6 (2.1)	8.9 (2.0)	0.05
Insulinogenic Index	1.15 (1.9)	1.17 (1.9)	0.9

Data presented as mean (sd) or as ^a^median (interquartile range). For continuous variables P was calculated by two-tailed t test and for categorical variables P was calculated by chi square test or Fisher’s exact test. Two women from the GDM group only had fasting blood samples taken at the follow up visit. Eleven samples of fasting glucose were coagulated and rejected by the department investigating glucose. *BMI* Body Mass Index, *BP* Blood pressure, *GA* Gestational age, *HOMA-IR* Homeostatic model of insulin resistance

### Influence of gestational diabetes on the salivary microbiota during pregnancy

**Fig. 1:**
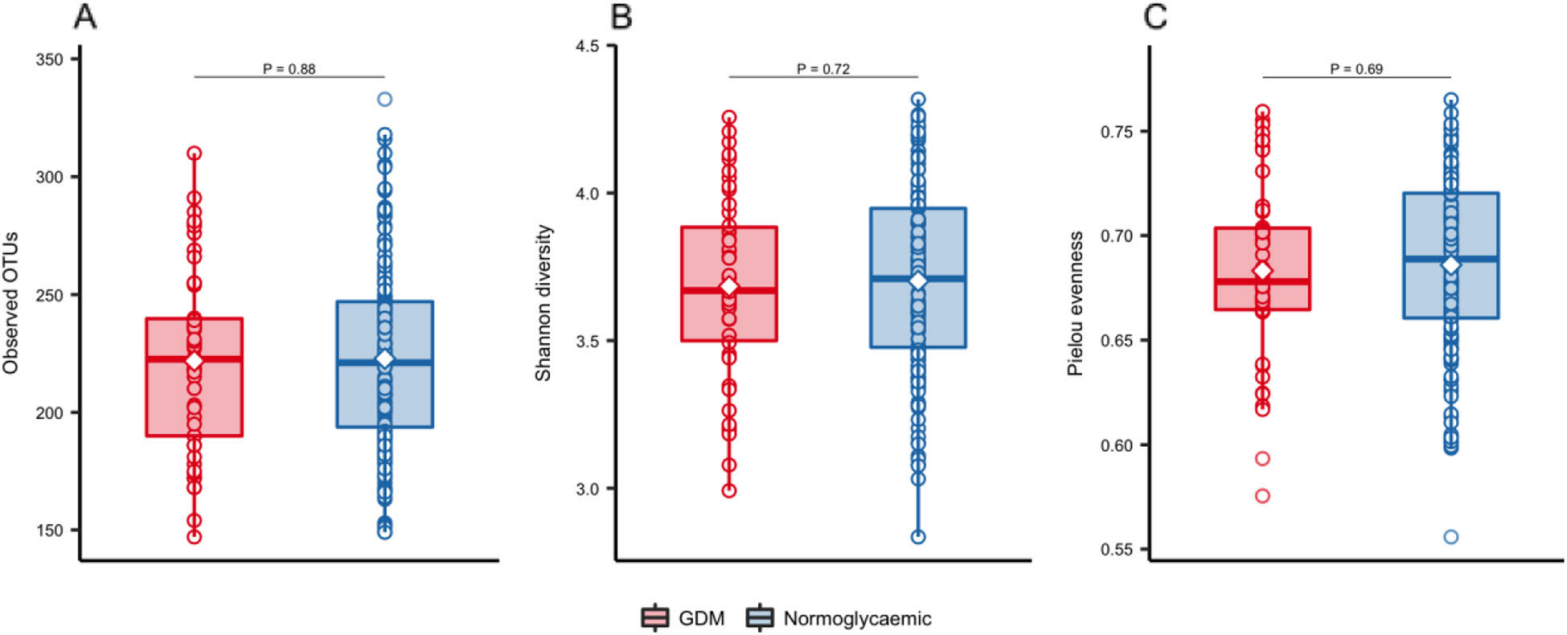
Alpha diversity of salivary microbiota during pregancy. Samples were rarefied to an equal sequencing depth of 10,000 reads and by observed OTUs (**a**), Shannon’s diversity (**b**) and Pielou’s evenness (**c**) alpha diversity in 3rd trimester of pregnancy in women with gestational diabetes (GDM; *n* = 50) and normoglycaemic women (*n* = 160) is presented. Boxes represent interquartile range (IQR), with the inside line representing the median. Values within 1.5 × IQR of the first and third quartiles are represented by whiskers. Circles represent individual samples with lines connecting samples from the same individual. Student’s t-test was used to test differences between normoglycaemic and GDM pregnancies

**Fig. 2:**
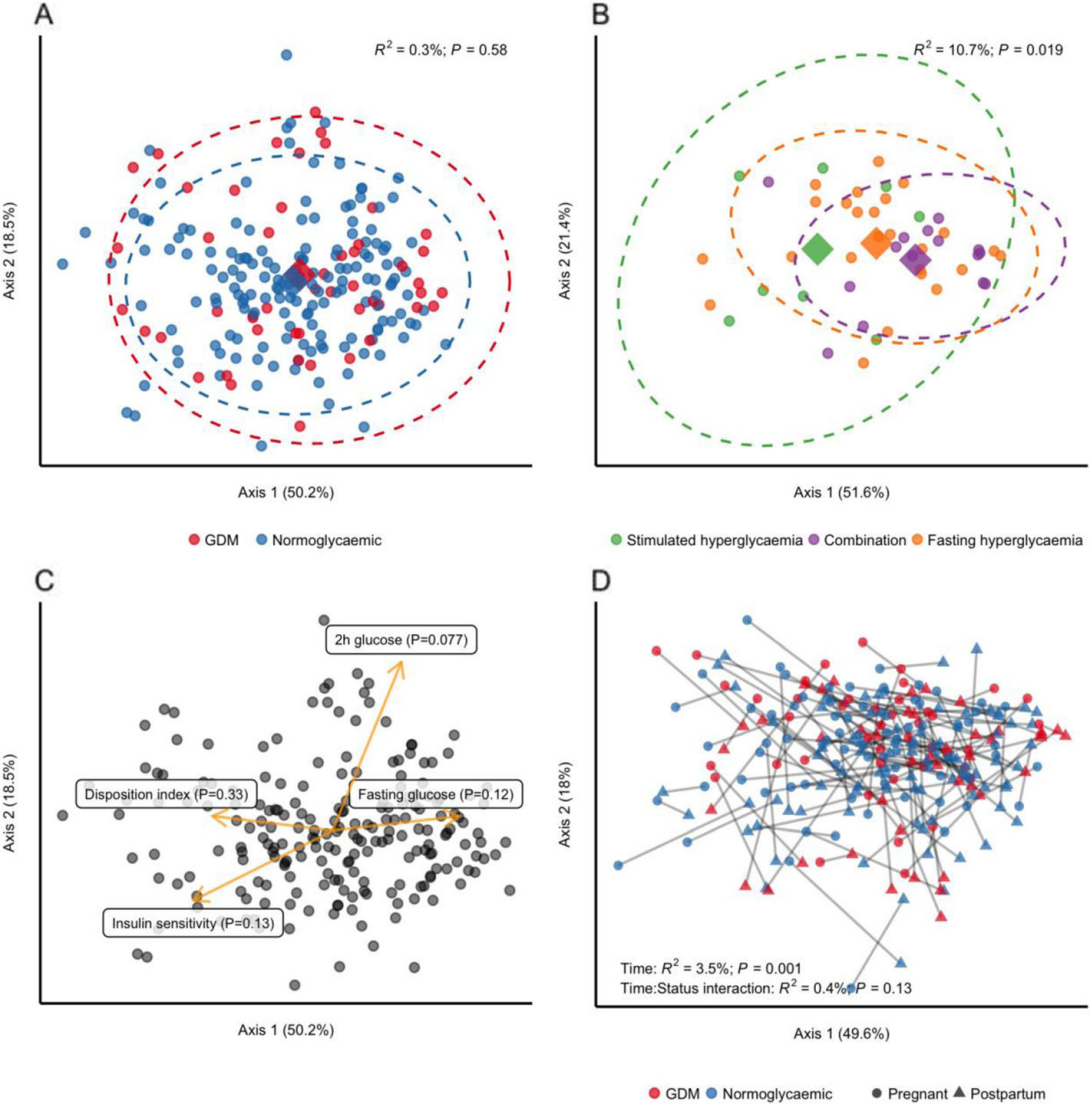
Community structure in women with or without gestational diabetes**.** For all analyses samples were rarefied to an equal sequencing depth of 10,000 reads prior to principal coordinate (PCo) ordination based on weighted Unifrac distances. **a** Samples from pregnant women in the 3rd trimester with (*n* = 50) or without (*n* = 160) gestational diabetes. Points are individual samples and diamonds represent the average ordination scores and ellipses the 95% confidence intervals of a multivariate normal distribution of either group. R2 and P are from permutational multivariate analysis of variance (PERMANOVA). **b** Community structure in pregnant women with gestational diabetes diagnosed by fasting hyperglycaemia (*n* = 25) or stimulated hyperglycaemia (2 h after an oral glucose challenge; *n* = 8), respectively, or a combination of the two (*n* = 15). Configuration is similar to panel A. **c** The association between glycaemic traits and community structure during pregnancy regardless of GDM status as determined by PERMANOVA. Vectors representing direction and magnitude of each trait were fitted onto the 1st and 2nd PCo axes using the envfit function of the vegan R package. **d** Change in community structure from pregnancy to postpartum. Only samples from women examined at both time points are included (*n* = 43 and *n* = 81 for women with and without GDM respectively). R2 and P are from PERMANOVA testing for a difference in community structure between samples collected during the 3rd trimester and those collected postpartum and for a differential change in community structure in women with GDM compared to women without GDM

We found no difference in richness (observed OTUs), overall diversity (Shannon’s index) or evenness (Pielou’s index) between women with GDM and women with normal gestational glucose regulation at a rarefied sequencing depth of 10,000 reads (Fig. 1). Similarly, we found no difference in community structure represented by weighted UniFrac distances (R^2^ = 0.3%, *P* = 0.58, Fig. 2a). The diagnosis of GDM was based on the presence of fasting or stimulated hyperglycaemia, or a combination of the two. Because fasting and stimulated hyperglycemia reflect two different pathophysiological states [31], we tested whether community structure differed in women diagnosed with GDM by different criteria. We found a significant difference in community structure with diagnostic category explaining 10.7% (*P* = 0.019, Fig. 2b) of the variation in weighted UniFrac distances. However, when testing the association between fasting and stimulated glucose concentration and community structure regardless of GDM status, the relationships were non-significant (Fig. 2c). Nor did we find an association between community structure and dynamic estimates of insulin sensitivity (Matsuda index; *P* = 0.13) or beta-cell function (Disposition index; *P* = 0.33).

**Fig. 3:**
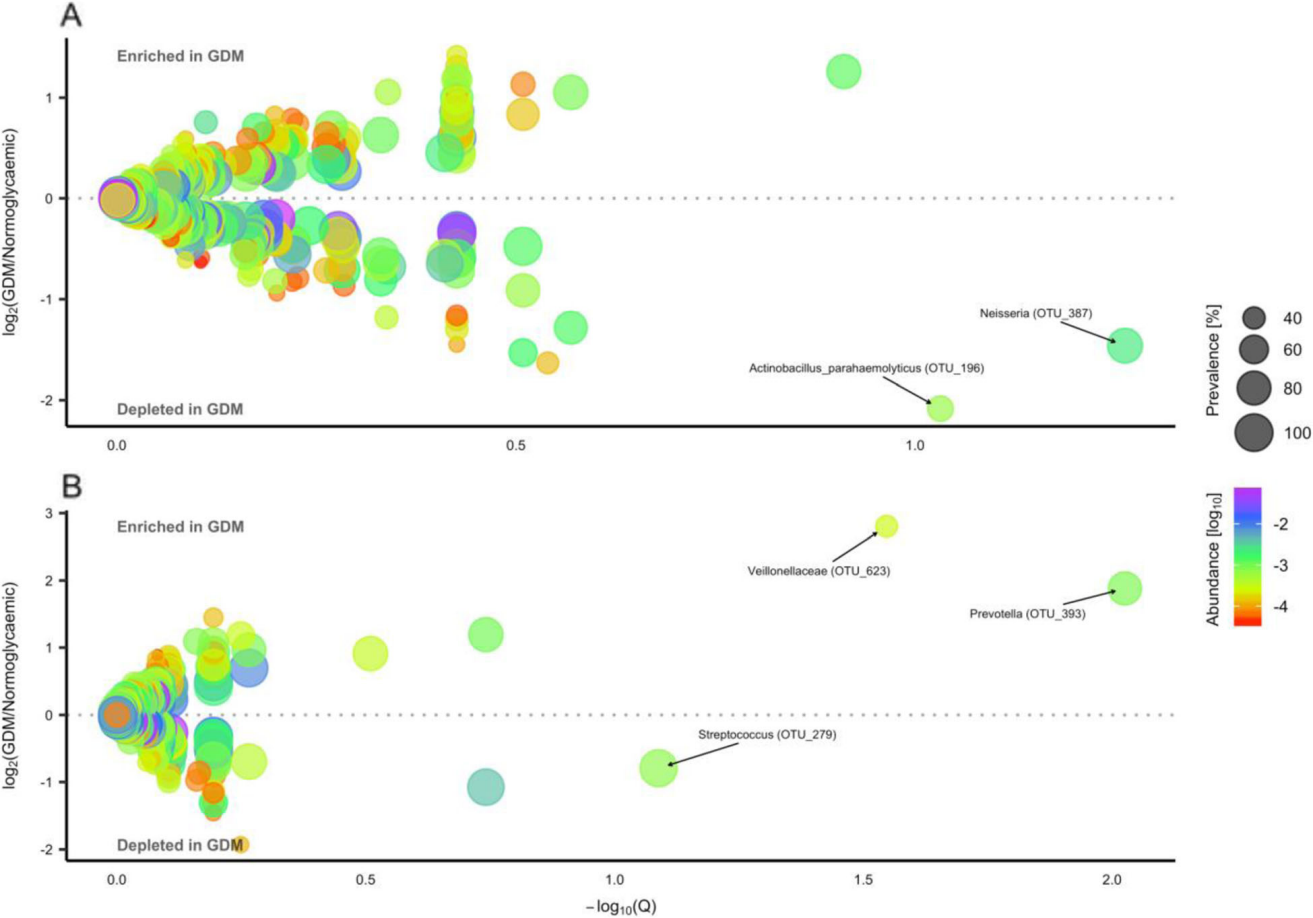
Operational taxonomic units associated with gestational diabetes during the third trimester with and without adjustment for pre-pregnancy bodymass index. **a** Estimated log_2_ fold difference in operational taxonomic unit (OTU) abundance between women with gestational diabetes (GDM, *n* = 50) and women with normal gestational glucose regulation (*n* = 160) during pregnancy and corresponding Benjamini-Hochberg adjusted *P*-values (Q) from the DESeq2 R package. Abundance is mean relative abundance of a given OTU. Prevalence is percentage of participants in which a given OTU is detected. Names of OTUs differentially abundant at a 10% false discovery rate are given at the lowest annotated taxonomic level. **b** Adjusted for pre-pregnancy body mass index in subset of women with available data (GDM, *n* = 43; normoglycaemic, *n* = 144)

In the third trimester of pregnancy and postpartum the main phyla of the salivary microbiota were *Firmicutes* and *Bacteroidetes* followed by *Proteobacteria*, with *Prevotella*, *Veillonella, Streptococcus* and *Haemophilus* as the predominant genera (Additional file 2: Figure S1, S2). Using negative binomial regression, we identified two OTUs which were less abundant in women with GDM in the third trimester, one assigned to *Actinobacillus paraheamolyticus* (OTU_196) and another to *Neisseria* (OTU_387), both within phylum *Proteobacteria* (Fig. 3a, Additional file 1: Table S4). When adjusting for pre-pregnancy BMI in the subset of women with available data (GDM = 43, normoglycaemic = 144) the above-mentioned associations were abolished, but three other differentially abundant OTUs were identified; one assigned to *Veillonellaceae* (OTU_623) and another assigned to *Prevotella* (OTU_393) were enriched, whereas one assigned to *Streptococcus* (OTU_279) was depleted in women with GDM (Fig. 3b, Additional file 1: Table S4). No genera were differentially abundant in women with GDM after adjustment for pre-pregnancy BMI.

### Association of microbial composition with glycaemic traits

When examining the relationship between microbial composition and glycaemic traits irrespective of GDM status, we identified *Treponema*, *Alloscardovia*, *Dialister*, *Filifactor*, *Parvimonas* and *Atopobium* as positively associated with 2-h stimulated plasma glucose. In contrast *Corynebacterium*, *Neisseria* and *Peptostreptococcus* were inversely associated with 2-h stimulated plasma glucose. Genus *Peptostreptococcus* was also positively associated with disposition index. Genera *Alloscardovia*, *Atopobium*, *Bulleidia*, *Dialister* and *Filifactor* were inversely associated with insulin sensitivity index (Additional file 1: Table S5, Additional file 2: Figure S3). No genera were associated with fasting plasma glucose.

To further identify species-level OTUs that are differentially abundant in women with the poorest gestational glucose regulation and women with the best gestational glucose regulation, samples were divided into tertiles of each trait and the upper and lower tertiles were contrasted using a negative binomial Wald test. We found 36 significant associations (FDR 10%) between 29 unique OTUs and three glycaemic traits (Additional file 1: Table S6, Additional file 2: Figure S4). Six associations with fasting plasma glucose level, 18 associations with 2-h stimulated plasma glucose level and 12 associations with disposition index were identified. No OTUs were associated with insulin sensitivity index. Six OTUs were associated with more than one glycaemic trait. As the only OTU, an *Actinomycetales* species (OTU_71) associated with higher fasting plasma glucose level and low disposition index, whereas a *Streptococcus* species (OTU_246) was associated with lower fasting plasma glucose and lower disposition index. *Porphyromonas endodontalis* (OTU_42) and *Rs-045* (OTU_60) of the candidate phylum *TM7* associated with higher 2-h stimulated plasma glucose level and with lower disposition index. A *Neisseria* species (OTU_387) associated with lower fasting plasma glucose level and higher 2-h stimulated plasma glucose level, whereas a *Peptostreptococcus* species (OTU_135) associated with lower 2-h stimulated plasma glucose and higher disposition index (Additional file 2: Figure S4). When adjusting for pre-pregnancy BMI, no associations remained statistically significant.

### Influence of gestational diabetes on the salivary microbiota after pregnancy

Nine months after delivery the composition of the salivary microbiota was similar to the composition during pregnancy (Additional file 2: Figure S2B). However, eight species-level OTUs displayed differential abundance according to previous GDM status. Two OTUs, assigned to *Bacteriodales* (OTU_142) and *Treponema* (OTU_242) respectively, were enriched in women with preceding GDM, whereas species-level OTUs assigned to *Leptotrichia* (OTU_37), *Streptococcus* (OTU_183), *Neisseria* (OTU_387), unclassified *Bacteria* (OTU_76), *Weeksellaceae* (OTU_29) and *Atopobium* (OTU_382) were depleted in the saliva of women with previous GDM (Additional file 1: Table S4). No OTUs exhibited differential change from pregnancy to postpartum in women with previous GDM compared to women with previous normoglycaemic pregnancy.

**Fig. 4:**
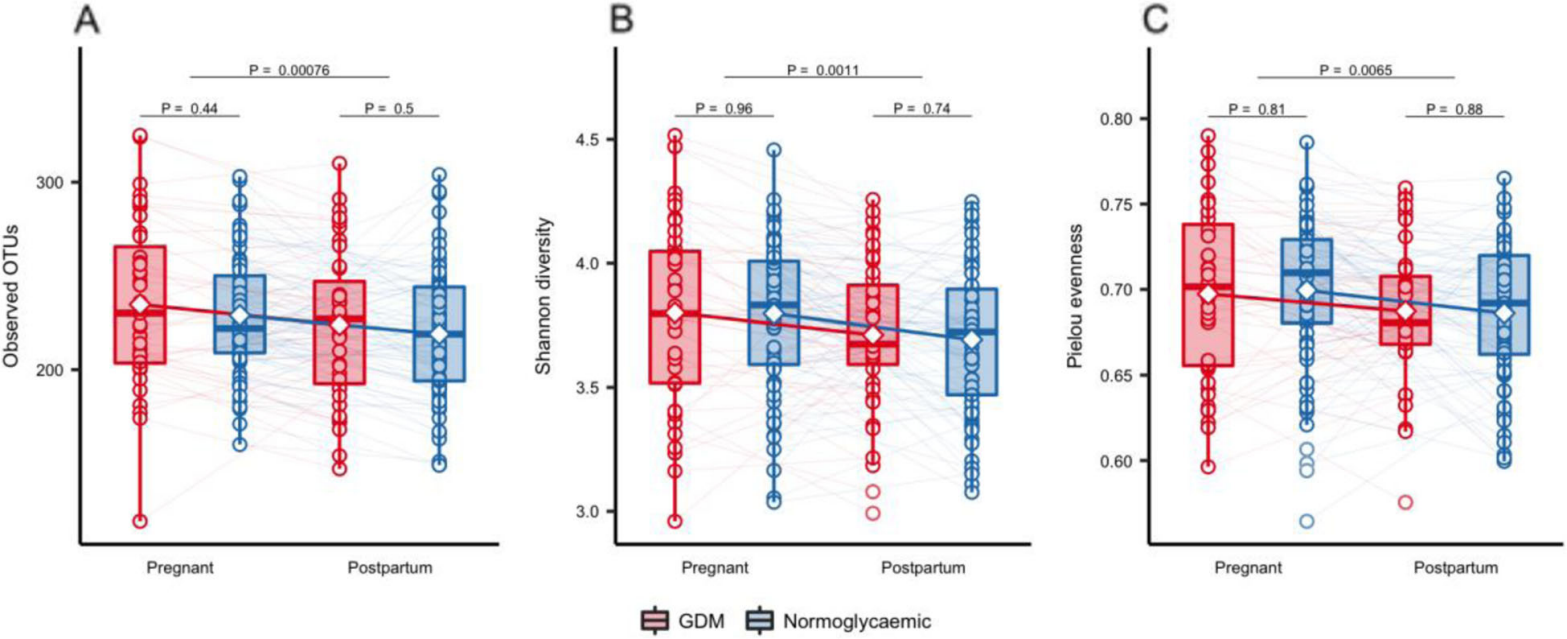
alpha diversity from third trimester of pregnancy to postpartum**.** Alpha diversity in pregnancy and postpartum as represented by observed richness (**a**), Shannon diversity (**b**) and Pielou evenness (**c**) based on samples from GDM (red, *n* = 43) and normoglycaemic (blue, *n* = 81) women with available faecal samples from 3rd trimester and 9 months postpartum. Samples were rarefied to an equal sequencing depth of 10,000 reads. Boxes represent interquartile range (IQR), with the inside line representing the median. Values within 1.5 × IQR of the first and third quartiles are represented by whiskers. Circles represent individual samples with lines connecting samples from the same individual. Students t-test were used to test differences between normoglycaemic and GDM pregnancies within each timepoint. Difference in richness, Shannon diversity and Pielou evenness between time points in GDM and normoglycaemic women combined was tested using a paired t-test

Accounting for the repeated nature of measurements performed on the same individual over time using mixed linear regression, we found that richness (number of observed OTUs), Shannon’s diversity and Pielou’s evenness decreased from pregnancy to postpartum (*P* = 0.0008, *P* = 0.001, *P* = 0.007 respectively; Fig. 4) in women with GDM and normal gestational glucose regulation alike. Similarly, we found a significant change in community structure (weighted UniFrac distances) from pregnancy to postpartum (R^2^ = 3.5%, *P* = 0.001, Additional file 2: Fig. 2d).

## Discussion

Our study of pregnant women demonstrated that, at the time of diagnosis, GDM is only associated with a moderately altered composition of the salivary microbiota. However, 9 months after delivery, GDM during the preceding pregnancy was associated with an aberrant salivary microbiota composition.

We found that richness of the salivary microbiota decreased from pregnancy to 9 months after delivery. A previous study of salivary microbiota over the course of a healthy pregnancy found higher bacterial richness in early pregnancy compared with late pregnancy in the same pregnant women. When comparing the salivary bacterial richness to a matched non-pregnant control group of women the authors found richness in early pregnancy significantly higher than in non-pregnant women, but did not report on statistical significance when comparing richness in late pregnancy with non-pregnant women [32]. Our finding is somewhat in line with previous observations of increased richness of salivary microbiota in pregnancy when comparing with non-pregnant adults, although our study represents two different time points of samples from the same individuals, and the previous study [32] compared a group of pregnant women to a group of matched non-pregnant controls.

Compared to first trimester, late pregnancy is characterized by increased levels of proinflammatory cytokines and insulin resistance [33] and in our study plasma glucose levels were, not surprisingly, higher during pregnancy than postpartum which could be expected to be mirrored in an increased salivary glucose level, as it has been described in non-pregnant adults [34]. Increased levels of salivary glucose have been associated with decreased bacterial richness in adolescents and salivary bacterial count could predict high salivary glucose concentration (salivary glucose ≥1.0 mg/dL) more accurately than clinical characteristics such as BMI and fitness levels [35]. These results are somewhat surprising as glucose is an energy source for many oral bacteria, however, the authors of the study argued that oral hyperglycaemia changed the oral microbiota environment by salivary acidification. In studies of the human gut microbiota, richness has been found to decrease with increasing proinflammatory state in the human host [36, 37]. Both findings are opposite to our findings of declining richness from pregnancy to postpartum. Interestingly, we have previously shown that, in the same group of women studied here, gut microbiota richness was higher during pregnancy compared to 9 months postpartum [25]. Increased microbiota richness in saliva and the gut in third trimester of pregnancy suggests that the proinflammatory state may have a different influence on microbiota composition during pregnancy than in non-pregnant adults and that the physiological changes during pregnancy influence the microbial community in the host. We hypothesize that increased bacterial richness in late pregnancy is of benefit for the fetus as it ensures that the child will encounter a more diverse bacterial ecosystem at birth. Comprehensive, longitudinal studies of women before, during and after birth, and their offspring are necessary to address this hypothesis.

So far, only one study has reported differences in the salivary microbiota composition during late pregnancy [38], and no differences has been reported postpartum in women with GDM compared with normoglycaemic women. An aberrant oral microbiota composition during pregnancy has been associated with adverse pregnancy outcome [16–20] and with type 2 diabetes in non-pregnant adults [6, 7]. The compositional aberration of the salivary microbiota during pregnancy and postpartum in women with GDM in our study has similarities with salivary microbiota previously described in non-pregnant individuals with type 2 diabetes. Species of *Neisseria* were depleted in women with GDM during pregnancy and postpartum. *Neisseria mucosa* has been reported to decrease in relation to an increased salivary glucose level, though not statistically significant [35]. In a mouse model of pregnancy, salivary *Neisseria* was found in the placenta of all animals, indicating that this species is able to migrate from the oral cavity to distant, anatomically separate organs [24]. This, together with our findings, indicates that decreased oral abundance of *Neisseria* potentially is associated with an unhealthy metabolic state in the human host and might have an influence on the increased risk of later metabolic disorders in children born to mothers with GDM. Similarly, a decreased abundance of oral *Atopobium* has been associated with an increased risk of diabetes [39] and in line with this we found *Atopobium* at the OTU-level to be depleted postpartum in women with previous GDM. The depletion of *Atopobium* postpartum in women with previous GDM might contribute to the known increased risk of developing type 2 diabetes [11].

Increased abundance of periodontal pathogens including *Porphyromonas gingivalis* and *Treponema denticola* has previously been reported in the oral microbiota of individuals with pre-diabetes [6] and type 2 diabetes [40–43]. *P. gingivalis* induces insulin resistance when administered orally in mice [8] and *T. denticola* and *T. socranskii* are positively associated with increased salivary glucose levels [35] and have been detected in increased abundance in the placenta of women with preeclampsia [44]. Postpartum we identified one *Treponema* OTU increased in women with previous GDM. Comparing non-pregnant and pregnant women, increased abundance of *Porphyromonas gingivalis*–a species with known immunomodulatory capacities [45, 46]–in subgingival plaque has been reported during pregnancy [32]. Interestingly, *P. gingivalis* has been detected in the amniotic fluid of women at risk of premature delivery [47] and in the placenta of pregnant women with preeclampsia [48, 49]. Based on these findings it could be speculated that *Porphyromonas* is implicated in the increased risk of adverse pregnancy outcomes associated with GDM.

Although not differentially abundant between women with and without GDM, we identified another species of *Porphyromonas, P. endodontalis*, which was positively associated with higher fasting and higher 2-h stimulated plasma glucose level and with lower disposition index. Like *P. gingivalis, P. endodontalis* is considered a periodontal pathogen [50], and we therefore speculate, that *P. endodontalis* might have similar effects on glucose homeostasis as *P. gingivalis* during pregnancy. Collectively, our findings suggest similarities between women with previous GDM and non-pregnant adults with periodontitis, pre-diabetes and type 2 diabetes. Likewise, *Streptococcus* spp. were depleted during pregnancy and postpartum in women with GDM, and during pregnancy abundance of different *Streptococcus* spp. were associated with lower fasting plasma glucose concentration, higher stimulated glucose and both lower and higher disposition index. *Streptococcus* in saliva has previously been positively correlated with cardiovascular disease markers in patients with atherosclerosis [51]. Our divergent results might reflect different strains of *Streptococcus*, but the applied 16S rRNA gene sequencing method does not allow us to investigate this at a higher taxonomic resolution.

Obesity has been found to alter the oral microbiota in non-pregnant women [52], and increased abundance of *Neisseria mucosa* and *Prevotella* has previously been reported in overweight non-pregnant adults compared with normal weight controls [52, 53]. After adjustment for pre-pregnancy BMI we found *Prevotella* enriched in pregnant women with GDM, and–without adjustment for BMI–*Prevotella* spp. were associated with higher concentrations of fasting and stimulated plasma glucose levels. Oral *Prevotella* has previously been associated with increased glucose concentration in women with GDM compared to healthy pregnant women at the time of delivery [38]. The same study found that the most remarkably concordance in microbial composition was between amniotic fluid and the oral microbiota in women with GDM, and the authors indicated that the that the microbial composition and variation of both mother and newborn could be driven by the health status of the pregnant woman and furthermore that the effects of GDM on microbes in pregnancy might be vertically transmitted to the baby during pregnancy [38].

Notably, we found that body mass index had a major influence on the salivary microbiota composition, as many of our results were abolished after adjustment for pre-pregnancy BMI. The strong confounding effect of BMI in our study suggests, that the variation in salivary microbiota composition associated with gestational diabetes and glycaemic traits during pregnancy, is related to the pre-pregnancy metabolic state of the host.

Uniquely, our study compares salivary microbiota composition in late pregnancy and 9 months postpartum in the same women making it possible to investigate pregnancy-associated changes in the salivary microbiota. However, the normoglycaemic control group might not be representative of pregnant women in general without any risk factors for development of GDM. Being at increased risk of developing GDM, our normoglycaemic women might have an aberrant salivary microbiota. Consequently, the difference between the two groups of women might not be as pronounced as if a group of healthy, pregnant women without risk factors had been studied. Another limitation of the study is that the periodontal status of the participants was not applicable in the study design, but would be of interest to investigate in a similar study.

## Conclusion

In conclusion, GDM is associated with a minor aberration of the salivary microbiota during late pregnancy and postpartum, and the composition of the salivary microbiota in women with GDM has similarities with salivary microbiota composition found in non-pregnant adults with type 2 diabetes. Regardless of GDM status, the bacterial content of the saliva is associated with glycaemic traits indicating that host physiology and a glucose-rich environment in the oral cavity may influence the presence and abundance of bacterial species. However, we reported a strong confounding effect of pre-pregnancy BMI indicating that body composition has an effect on the composition of salivary microbiota. Richness of the salivary microbiota is higher during pregnancy compared with postpartum, which might be mediated by immunological changes.

## Supplementary information

**Additional file 1: Table S1.** Supplementary third trimester characteristics between pregnant women with GDM and glucose-tolerant pregnant women. **Table S2.** Indications for referral to an oral glucose tolerance test. **Table S3.** Supplementary descriptive postpartum. **Table S4.** Differentially abundant OTUs during pregnancy and postpartum between women with or without GDM. **Table S5.** Association between genera and glycaemic traits in third trimester of pregnancy. **Table S6.** Operational taxonomic units associated with glycaemic traits in pregnant women independent of GDM status and unadjusted for pre-pregnancy BMI.

**Additional file 2: Figure S1.** Individual phylum and genus level composition third trimester pregnant women. **Figure S2.** Individually phylum composition nine months postpartum. **Figure S3.** Bacterial genera associated with glycaemic traits in pregnant women regardless of GDM status. **Figure S4.** Heatmap of bacterial operational taxonomic units associated with glycaemic traits during pregnancy.

## Data Availability

The datasets generated and/or analyzed during the current study is not publicly available due to Danish Data Protection Agency but are available from the corresponding author on reasonable request.

## Abbreviations

16S rRNA: 16S ribosomal ribonucleic acid
BMI: Body Mass Index
GDM: Gestational Diabetes Mellitus
OGTT: Oral Glucose Tolerance Test
OTU: Operational Taxonomic Units
V1-V3: Variable regions of the 16S ribosomal RNA
