# Supplementary material for: Gestational diabetes and the human salivary microbiota: a longitudinal study during pregnancy and postpartum
Source: BMC Pregnancy Childbirth. 2020 Jan 31;20:69. doi: 10.1186/s12884-020-2764-y (PMC6995204; doi:10.1186/s12884-020-2764-y)
Supplement: Supplementary file 2 — Additional file 2: Figure S1. Individual phylum and genus level composition third trimester pregnant women. Figure S2. Individually phylum composition nine months postpartum. Figure S3. Bacterial genera associated with glycaemic traits in pregnant women regardless of GDM status. Figure S4. Heatmap of bacterial operational taxonomic units associated with glycaemic traits during pregnancy. [file 12884_2020_2764_MOESM2_ESM.pdf]

Supplementary figures

Figure S1: Individual phylum and genus level composition third trimester pregnant women

A

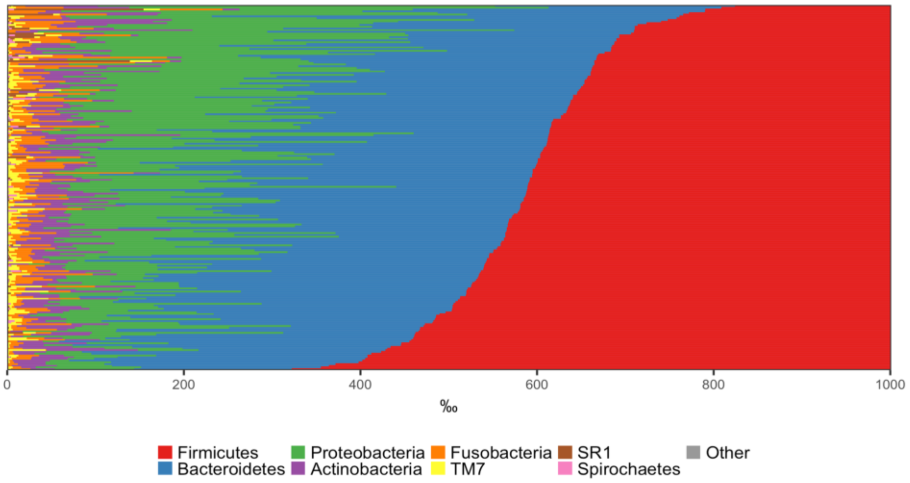

B

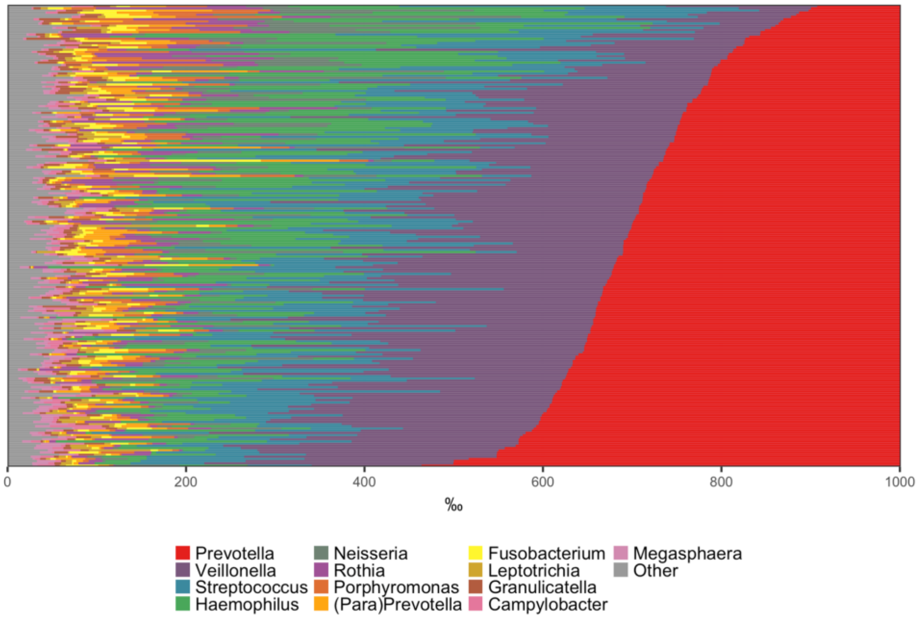

Mean read count of individually composition at phylum [A]and genus [B] level in third trimester of pregnancy

Figure S2: Individually phylum composition nine months postpartum

A

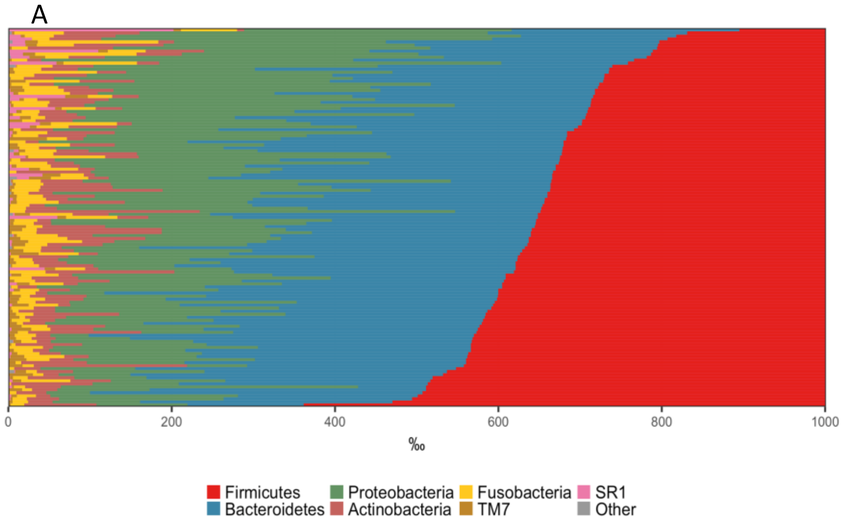

B

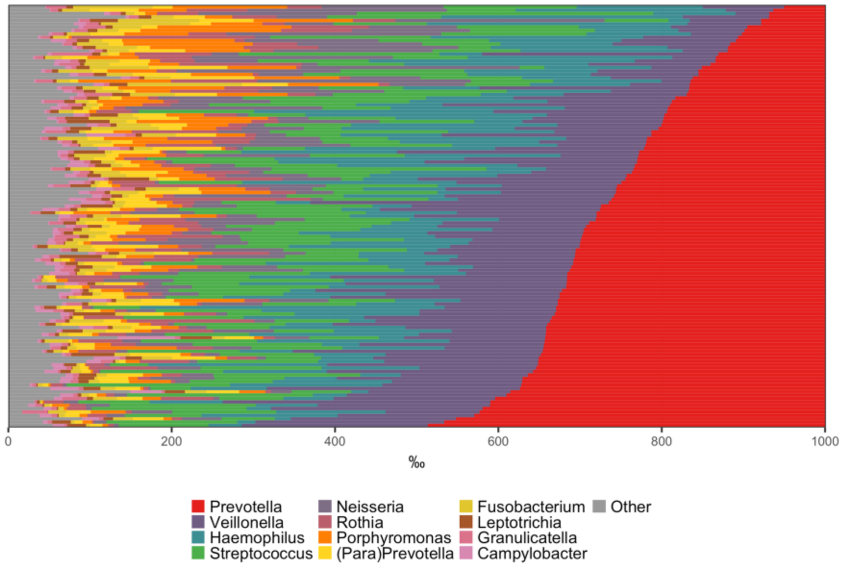

Composition of the salivary microbiota nine monhts postpartum at individually phylum [A] and genus [B] level

Figure S3: Bacterial genera associated with glycaemic traits in pregnant women regardless of GDM status

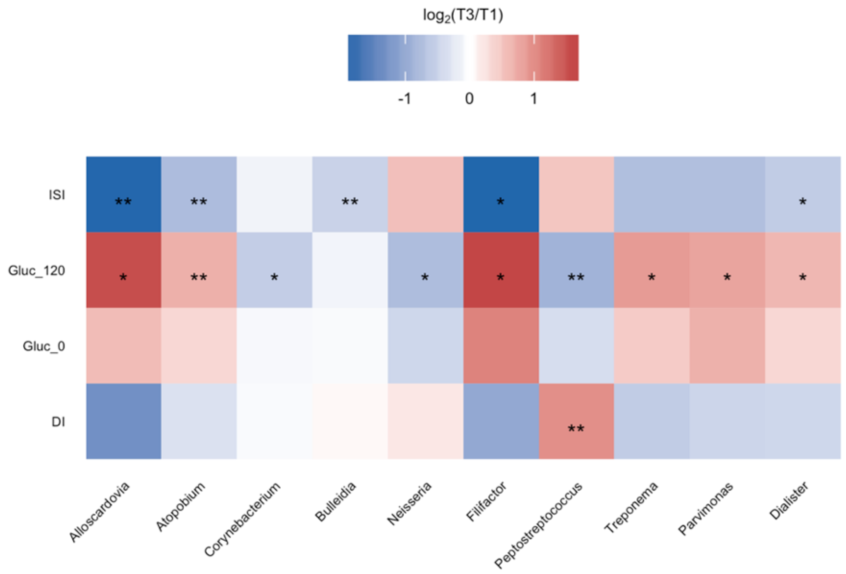

Heatmap of correlations between bacterial genera and fasting plasma glucose, stimulated 2h glucose, insulin sensitivity, and disposition index, without adjustment for body mass index. Only taxa nominally associated with either of the four traits are depicted. Taxa are ordered taxonomically. Results for all taxa are presented in table S5. * Q<=0.1. ** Q<=0.05

Figure S4: Heatmap of bacterial operational taxonomic units associated with glycaemic traits during pregnancy

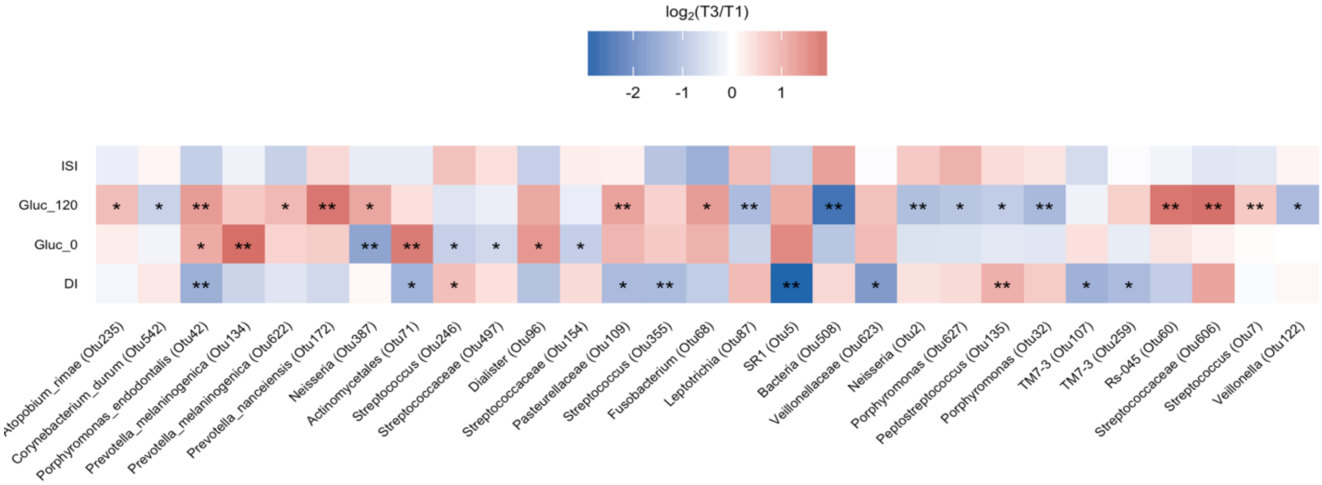

Samples were divided into tertiles based on fasting plasma glucose, stimulated 2h glucose, insulin sensitivity, and disposition index respectively. The plot depicts the $\log_2$ fold difference in operational taxonomic unit (OTU) abundance between women in the upper tertile (T3) compared to the lower tertile (T1) of each glycaemic trait tested using the negative binomial Wald test implemented in the DESeq2 R package. Only OTUs significantly associated with either of the four traits at a 10% false discovery rate are depicted. Names are given at the genus level. OTUs are ordered alphabetically by genus annotation. Results in tabular form are available in Table S6. * Q<=0.1 ** Q<=0.05
